# Combining Cochlear Analysis and Auditory Evoked Potentials in a Beluga Whale With High-Frequency Hearing Loss

**DOI:** 10.3389/fvets.2020.534917

**Published:** 2020-11-04

**Authors:** Maria Morell, Stephen A. Raverty, Jason Mulsow, Martin Haulena, Lance Barrett-Lennard, Chad A. Nordstrom, Frederic Venail, Robert E. Shadwick

**Affiliations:** ^1^Zoology Department, The University of British Columbia, Vancouver, BC, Canada; ^2^INSERM Unit 1051, Institute for Neurosciences of Montpellier, Montpellier, France; ^3^Institute for Terrestrial and Aquatic Wildlife Research, University of Veterinary Medicine Hannover, Foundation, Büsum, Germany; ^4^Animal Health Center, Ministry of Agriculture, Abbotsford, BC, Canada; ^5^National Marine Mammal Foundation, San Diego, CA, United States; ^6^Vancouver Aquarium Marine Science Center, Vancouver, BC, Canada; ^7^Coastal Ocean Research Institute, Vancouver Aquarium, Vancouver, BC, Canada; ^8^Fisheries and Oceans Canada, Pacific Biological Station, Nanaimo, BC, Canada

**Keywords:** inner ear, cochlea, auditory evoked potentials, beluga, scanning electron microscopy, immunofluorescence, high-frequency hearing loss, *Delphinapterus leucas*

## Abstract

Correlations between inner ear morphology and auditory sensitivity in the same individual are extremely difficult to obtain for stranded cetaceans. Animals in captivity and rehabilitation offer the opportunity to combine several techniques to study the auditory system and cases of hearing impairment in a controlled environment. Morphologic and auditory findings from two beluga whales (*Delphinapterus leucas*) in managed care are presented. Cochlear analysis of a 21-year-old beluga whale showed bilateral high-frequency hearing loss. Specifically, scanning electron microscopy of the left ear revealed sensory cell death in the first 4.9 mm of the base of the cochlea with scar formation. Immunofluorescence microscopy of the right ear confirmed the absence of hair cells and type I afferent innervation in the first 6.6 mm of the base of the cochlea, most likely due to an ischemia. Auditory evoked potentials (AEPs) measured 1.5 years prior this beluga's death showed a generalized hearing loss, being more pronounced in the high frequencies. This individual might have had a mixed hearing loss that would explain the generalized hearing impairment. Conversely, based on AEP evaluation, her mother had normal hearing and subsequent cochlear analysis did not feature any apparent sensorineural pathology. This is believed to be the first study to compare two cochlear analysis techniques and hearing sensitivity measurements from AEPs in cetaceans. The ability to combine morphological and auditory data is crucial to validate predictions of cochlear frequency maps based on morphological features. In addition, our study shows that these three complementary analysis techniques lead to comparable results, thus improving our understanding of how hearing impairment can be detected in stranding cases.

## Introduction

Correlations between inner ear morphology and auditory sensitivity in the same individual are extremely difficult to obtain for cetaceans. Live strandings, animals in rehabilitation, and captive specimens are likely the only candidates that can provide such valuable information. Here, we present and compare the results of the inner ear analysis of 21-year-old (Qila) and 40-year-old (Aurora, Qila's mother) female beluga whales from the Vancouver Aquarium for which auditory evoked potential (AEP) thresholds were fortuitously measured 1.5 years prior to death.

Previous studies on hearing loss in humans and terrestrial mammals have demonstrated structural alterations of the organ of Corti (or hearing organ) and associated innervation. These lesions have been attributed to mechanical damage and metabolic fatigue of the sensory cells and associated with high-intensity and/or long-duration sound exposure (1), exposure to ototoxic drugs, genetic, infectious (bacterial and viral), and geriatric processes (2). Changes in the sensory cell stereocilia and degeneration and loss of the entire hair cell have been documented (3–5), as well as swelling of the afferent nerve endings on the inner hair cells with incipient retrograde nerve degeneration (6). In more severe cases, complete degeneration of the organ of Corti can occur (3). When a mammalian cochlear hair cell dies, the contiguous supporting cells actively participate in the process of hair cell elimination and scar formation. This “scarring” process comprises the simultaneous expansion and sealing of the reticular lamina (1) as a rapid protective response to hair cell apoptosis. Loss of hair cells in the organ of Corti in mammals is permanent and results in irreversible hearing impairment. Thus, the presence of scarring among hair cell rows is an important criterion to assess for any possible history of hearing loss. These scars can be differentiated from artifactual exfoliation of hair cells that occurs with autolysis (7, 8). In cases of a severe trauma that causes direct damage to the supporting cells, or repeated less severe lesions, supporting cells may not remain differentiated. When the differentiated supporting cells are absent, the basilar membrane is covered by a layer of simple squamous or cuboidal epithelia, termed “flat epithelium” (9). At present, it is unclear whether the flat epithelium represents de-differentiated supporting cells or cells that migrated from adjoining regions.

There is a lack of ultrastructural analysis or characterization of the organ of Corti in most cetacean species. However, the normal morphology of the cochlea and changes in the morphology of the sensory cells along the cochlear spiral has been recently documented in beluga whales (10). Correlations of different cochlear analysis techniques with AEP hearing sensitivity measurements have not, to the best of our knowledge, previously been reported for cetaceans. The ability to correlate morphological and auditory data is imperative to assess how underwater sound and other etiologies might impact hearing in wild cetaceans. This is especially important for individuals where the hearing impairment may be interpreted and inferred solely based on ultrastructural and histopathological studies, since live stranded animals are not always available for testing. With the documented increase in anthropogenic noise in the marine environment, it is critical to characterize and establish these baseline data. In addition, this correlation between cochlear anatomical and auditory data is crucial to validate predictions of cochlear frequency maps (i.e., frequency distribution along the cochlear spiral) based on morphological features. These maps are important for determining the frequency range that is impaired if lesions are found.

## Materials and Methods

### Auditory Evoked Potentials (AEPs)

AEP measurements were conducted on the belugas Qila and Aurora in March 2014. The AEPs were elicited using an acoustic projector embedded in a suction cup (termed a “jawphone”) placed on the lower jaw near the commissure of the mouth (left side only for Aurora, left and right for Qila). Although there is some degree of acoustic crosstalk between the two ears with this arrangement, it is likely that the AEPs are dominated by activity from by the ear closest to the jawphone, especially at threshold (11–13). Therefore, the auditory brainstem responses (ABRs) in this study are designated as arising from stimulation of the “left” and “right” ears, although the ABRs almost certainly include contributions from the contralateral ear to the jawphone. The jawphone comprised an International Transducer Corporation 1042 piezoelectric transducer embedded in a silicone suction cup. This type of projector is commonly used in AEP threshold audiometry with odontocete cetaceans, and threshold data obtained with jawphones produce audiograms that are comparable, especially in shape and high-frequency hearing limit, to those obtained underwater in a direct field (14). Acoustic stimulus production and AEP recording were conducted using the Evoked Response Study Tool [EVREST, (15, 16)] software and a ruggedized laptop computer.

**Figure 1:**
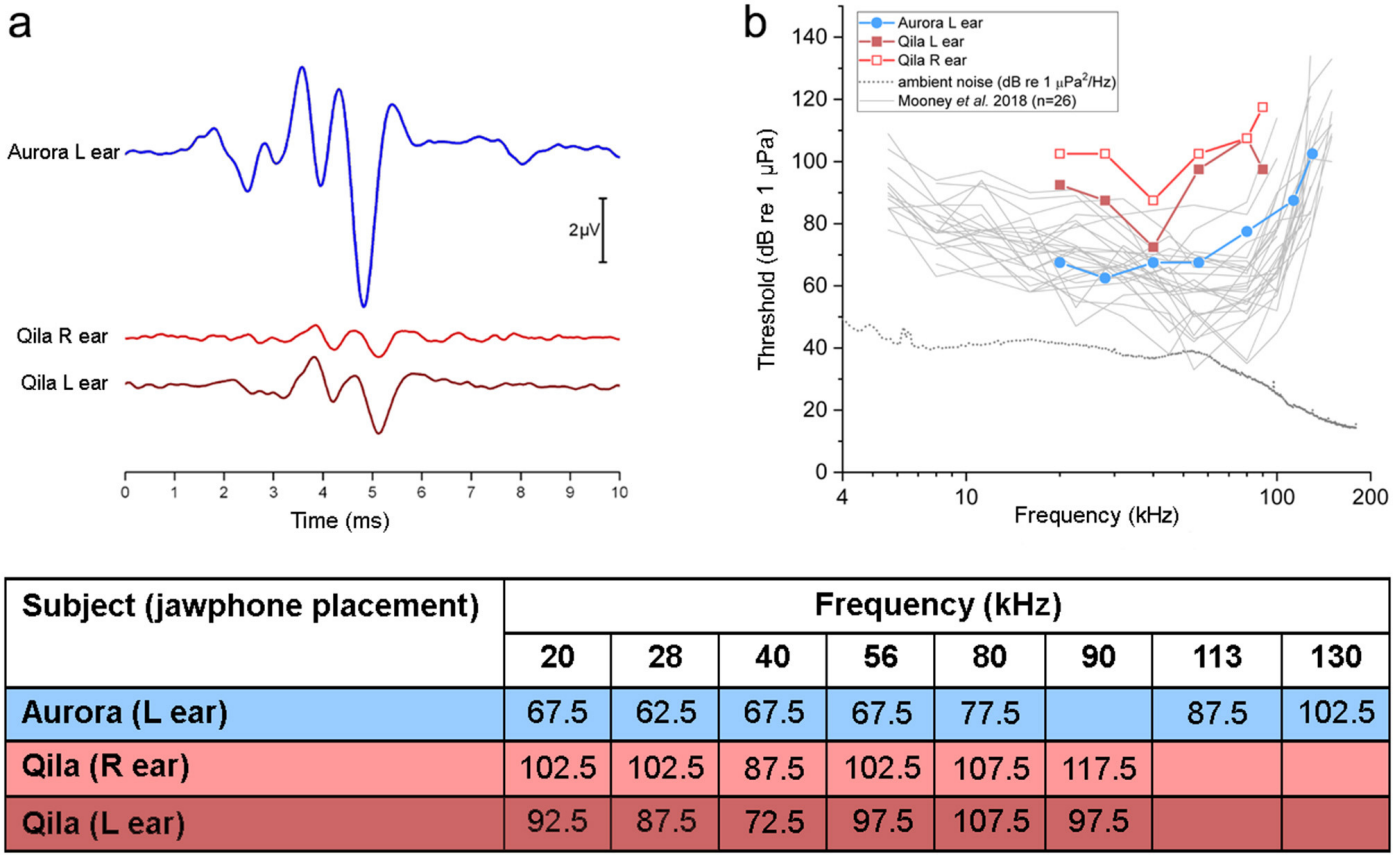
**(a)** Click-evoked auditory brainstem responses (ABRs) measured for the 40-year-old beluga Aurora (blue) and 21-year-old Qila (red). ABRs from Aurora were of relatively large amplitude. In contrast, ABRs from both ears from Qila had smaller amplitude, with increased latencies of the dominant waves. **(b)** Auditory evoked potential audiograms measured in Aurora (left ear, blue circle) and Qila (left ear, red closed squares; right ear, red open squares), in comparison with those reported by Mooney et al. (25) for 26 belugas. Underwater ambient noise levels (power spectral density, units of 1 μPa^2^/Hz) for the pool in which Aurora and Qila were tested are shown with a dotted line. Note that Qila has a hearing loss which is most apparent in the high frequencies at 56 kHz and above (see table) and in the right ear (light red). Table in **(b)** AEP hearing thresholds (in dB re 1 μPa) for Aurora and Qila tested in March 2014. Hearing threshold was defined as the mid-point between the lowest stimulus level for which a statistically significant response was detected, and the highest level for which no AEP was detected. R, right; L, left.

The hearing tests took place in a medical pool, where the floor was raised such that the belugas were resting on the pool floor with their dorsal surface above the water. Ambient noise levels measured in the pools were ~40 dB re 1 μPa^2^/Hz from 10–60 kHz, and progressively decreased to ~15 dB re 1 μPa^2^/Hz at 180 kHz (Figure 1b). The jawphone was underwater on the lower jaws near the oral commissures. The belugas were temporarily restrained by the Vancouver Aquarium training staff, and monitored by attending veterinarians during data collection. Small stainless-steel subdermal electrodes (Grass Technologies, 12 mm × 30 gauge) were placed on the dorsal surface for recording of the ongoing electroencephalogram (EEG). A non-inverting electrode was placed ~5–10 cm behind the blowhole. A ground electrode was placed on the back near the dorsal fin, and an inverting electrode was placed midway between the inverting and ground electrodes. The EEG was amplified (100 dB) and bandpass filtered (0.3–3 kHz) using a Grass Technologies ICP511 biopotential amplifier. The analog EEG signal was digitized at a rate of 50 kHz using a National Instruments PCI-6251 data acquisition card and saved to hard disk for later analysis.

Prior to frequency-specific testing, transient click-evoked AEPs (i.e., ABRs) were elicited using a 25-μs pulse (1 MHz digital-to-analog conversion rate) sent to the jawphone. The resulting “click” featured energy in the frequency region where belugas are sensitive, with a peak frequency near 100 kHz [see (11) for more details of the stimulus]. The click level was estimated at 153 dB re 1 μPa (peak-equivalent sound pressure level). Frequency-specific thresholds were subsequently obtained by measuring the auditory-steady state response (ASSR, also called the envelope following response), a rhythmic AEP that can be evoked by a sinusoidal amplitude-modulated (SAM) tonal stimulus (17, 18). The SAM tones were 22 ms in duration (1-ms rise/fall, cosine window), with center frequencies ranging from 20 to 130 kHz and 100% amplitude modulation at a rate of 1 kHz, and levels calibrated in dB re 1 μPa, root-mean-square. The stimulus levels for both the clicks and SAM tones were calibrated using a receiving hydrophone placed underwater, 15 cm from the main transmitting axis of the jawphone suction cup. It should be noted that because the recorded levels are only rough estimates of those actually received by the belugas, the threshold values should be considered estimates as well. Relative changes between audiogram shapes, especially with regards to the upper-frequency hearing limit are reliable, however.

The clicks and SAM tones were both delivered to the belugas at a rate of ~31 ms^−1^. Final records were generated by averaging the EEG time-locked to 1,024 presentations of the stimuli, with the polarity of stimuli alternating on successive presentations to reduce electrical artifacts in the records.

Thresholds for the frequency-specific SAM tones were determined by first conducting a fast Fourier transform (FFT) of 20 ms of the averaged AEP, starting 4 ms after stimulus onset. The amplitudes of the resulting responses were determined by measuring the peak of the AEP spectrum at 1 kHz [i.e., the AEPs followed the rate of stimulus amplitude modulation, see (18)]. Stimulus levels were successively attenuated at each frequency until no statistically significant peak was detected in the response spectrum [magnitude-squared coherence test, α = 0.01, (19, 20)]. Threshold was defined as the average of the lowest stimulus level with a significant response and the highest level without a significant response.

All animal procedures and animal research were approved by the Vancouver Aquarium Institutional Animal Care and Welfare Committee.

### Ear Analysis

The 21-year-old beluga whale Qila died on November 16th, 2016, at the Vancouver Aquarium Vancouver, British Columbia and the ears were collected ~5.5 h *post-mortem*. Her mother (40-year-old) Aurora succumbed on November 25th 2016, and her ears were fixed ~16 h *post-mortem*. According to a previous optimized protocol (21), the inner ears from both individuals were perfused perilymphatically with 10% neutral buffered formalin and the left tympanic bone from Qila was processed for histopathology. For both belugas, the left cochlea was evaluated by scanning electron microscopy (SEM) and the right cochlea was processed for immunofluorescence, using specific antibodies to label sensory cells and their associated innervation. Conventional and validated protocols were used for sample processing for SEM and immunofluorescence (7, 8, 22). In addition, a few subsections of the left inner ear from Aurora were processed for histopathology.

#### Decalcification

The periotic bones were decalcified by immersion in 14% Ethylenediaminetetraacetic acid (EDTA) tetrasodium salt hydrate, pH 7.4, at room temperature [changing the solution once every 7–10 days (23)] during 57–61 days in both individuals. The tympanic bone from Qila was decalcified with the rapid decalcifier RDO® (Apex Engineering Products Corporation, Aurora, Illinois, US) for 3 days.

#### Histology

After decalcification, the tympanic bone of Qila and the round window niche and surrounding tissue of Aurora were processed by conventional histology techniques with an automated processor (TissueTek). Tissues were embedded in paraffin, sectioned at 5 μm, and stained with Hematoxylin and Eosin, periodic acid Schiff's (PAS) and Twort's Gram stain.

#### Scanning Electron Microscopy

The decalcification of the left periotic bones ceased when the vestibular scala and the *stria vascularis* of the cochlea were exposed. Subsequently, the cochleas were dissected, dehydrated with increasing concentrations of ethanol, critical point dried with CO_2_, and then coated with gold-palladium. The samples were observed with an SEM at the University of British Columbia (UBC) Bioimaging Facility, Canada (Hitachi S-4700). Brightness and contrast of the figures were enhanced in Photoshop®.

#### Immunofluorescence

The right cochleas were dissected for immunofluorescence imaging using the whole-mount technique, following our recently optimized protocol (22). Preserved cochleas were transversely sectioned into four pieces and further dissected until flat preparations were obtained. The Reissner and tectorial membranes were removed, and the spiral ligament trimmed below the *stria vascularis*. The spiral ganglion cells were retained in most of the preparations, but the modiolus was removed.

The flat cochlear sections were initially blocked with 5% normal donkey serum for 1 h at room temperature, incubated with the primary antibodies overnight at 4°C, then with secondary antibodies for 2 h in the dark, and for 30 min with DAPI (Thermo Scientific ref. 62248, 1:1,000 dilution), to counter-stain the nucleus. The following primary antibodies were used: (1) goat anti-prestin polyclonal IgG antibody (Santa Cruz ref. SC-22692, 1:200), which labels the outer hair cells; (2) rabbit anti-myosin VI polyclonal antibody (Proteus ref. 25-6791, 1:500) that labels inner and outer hair cells, and (3) mouse anti-neurofilament 200 (phosphorylated and non-phosphorylated) monoclonal antibody IgG1 isotope (Sigma-Aldrich ref. N0142, 1:400), which labels type I afferent innervation that comprises 95% of the afferent innervation of the cochlea in some species of odontocetes (24). The secondary antibodies used were Alexa Fluor® 488 donkey anti-goat IgG, Alexa Fluor® 568 donkey anti-rabbit IgG and Alexa Fluor® 647 donkey anti-mouse IgG (Molecular Probes refs. A11055, A10042, A31571, respectively, 1:400).

Small subsections from the four large half turns were processed as controls. These included: (1) Specificity control (samples were incubated with the same concentration of IgG as the primary antibodies, and then incubated with the secondary antibodies and DAPI); (2) Control for non-specific binding of the secondary antibodies (samples were incubated without the primary antibodies, and then incubated with the secondary antibodies); (3) Control for autofluorescence (no primary and no secondary antibodies were used).

All fragments, including the controls, were treated with 0.2% Sudan Black B for 10 min after the secondary antibody to reduce the fluorescence of the tissue. The flat preparations were individually mounted on a glass slide with 0.1% N-propyl gallate in 90% glycerol and evaluated using fluorescence optic microscope and an Olympus FV1000 confocal microscope at the UBC Bioimaging Facility (Vancouver, Canada) and a Zeiss LSM880 confocal microscope at the Montpellier Resources Imagery (MRI, Montpellier Cell Biology Research Center, France). Micrographs of the three controls were obtained using the same magnification and same intensity of the four lasers as their respective treatments. Brightness and contrast of the figures were enhanced, using identical protocols for treatments and the respective controls.

## Results

### Auditory Evoked Potentials (AEPs)

Click-evoked ABRs were recorded prior to measuring frequency-specific hearing thresholds. These were used as a rapid, gross measure of auditory function, and to ensure that all equipment was functioning properly. The resulting ABRs are shown for the belugas Aurora (in blue in Figure 1a) and Qila (in red in Figure 1a). For Aurora, the peaks of the waveforms were of relatively large amplitude when stimuli were delivered from the left jaw (i.e., ABRs are dominated by activity from the left ear, peak-to-peak amplitude of 7.5 μV). However, the ABRs from both ears from Qila are of smaller amplitude (peak-to-peak amplitudes of 2.4 and 1.0 μV for the left and right ears, respectively). The latencies of the waves are also relatively delayed in the ABRs for Qila. For example, the latency to the dominant vertex-negative potential was 4.8 ms following acoustic stimulation in Aurora, as compared with 5.1 ms for both ears in Qila.

Following click-evoked ABR testing, hearing thresholds as a function of frequency (AEP audiograms) were measured for both belugas. The thresholds for Aurora, Qila, and 26 wild belugas previously tested using similar AEP methods (25) are shown in Figure 1b. This large sample size from Mooney et al. (25) bounds nearly all other AEP thresholds reported for belugas (17, 26, 27), and thus they are used to provide a reference to the normal values for this species and method [but see (28–32) for behavioral thresholds]. Aurora's thresholds were comparable to those for belugas from Mooney et al. (25) at all examined frequencies (blue and gray traces in Figure 1b). The hearing sensitivity of Qila was reduced relative to the other belugas (red in Figure 1b), and no frequency-specific AEPs could be detected above 90 kHz. Similar to the patterns observed for the click-evoked ABRs of Qila, hearing impairment was more apparent in the right ear (light red in Figure 1) than in the left ear (dark red in Figure 1); all thresholds for Qila's right side were higher than those reported for the other individuals.

### Ear Analysis

Full necropsies of both belugas were conducted at the Animal Health Center, Abbotsford, BC, ~100 km east of the Vancouver Aquarium. Post-mortem examination of Qila revealed an infection of *Nocardia paucivorans* within the lung with reactive change in regional lymph nodes. Based on the extent and severity of inflammation and necrosis, this infection would have contributed at least moderately to impaired respiratory function. The proximate cause of death could be attributed to hypovolemic shock in the case of Aurora, secondary to liver and caudolateral and retroperitoneal hemorrhage, peritoneal rent and hemoperitoneum. The presumptive liver atrophy and hemorrhage, desquamated epithelia within the esophagus and nonglandular compartment of the stomach are consistent with inappetence or anorexia and may be related to the preexisting pathologic processes.

#### Left Ear From Qila

**Figure 2:**
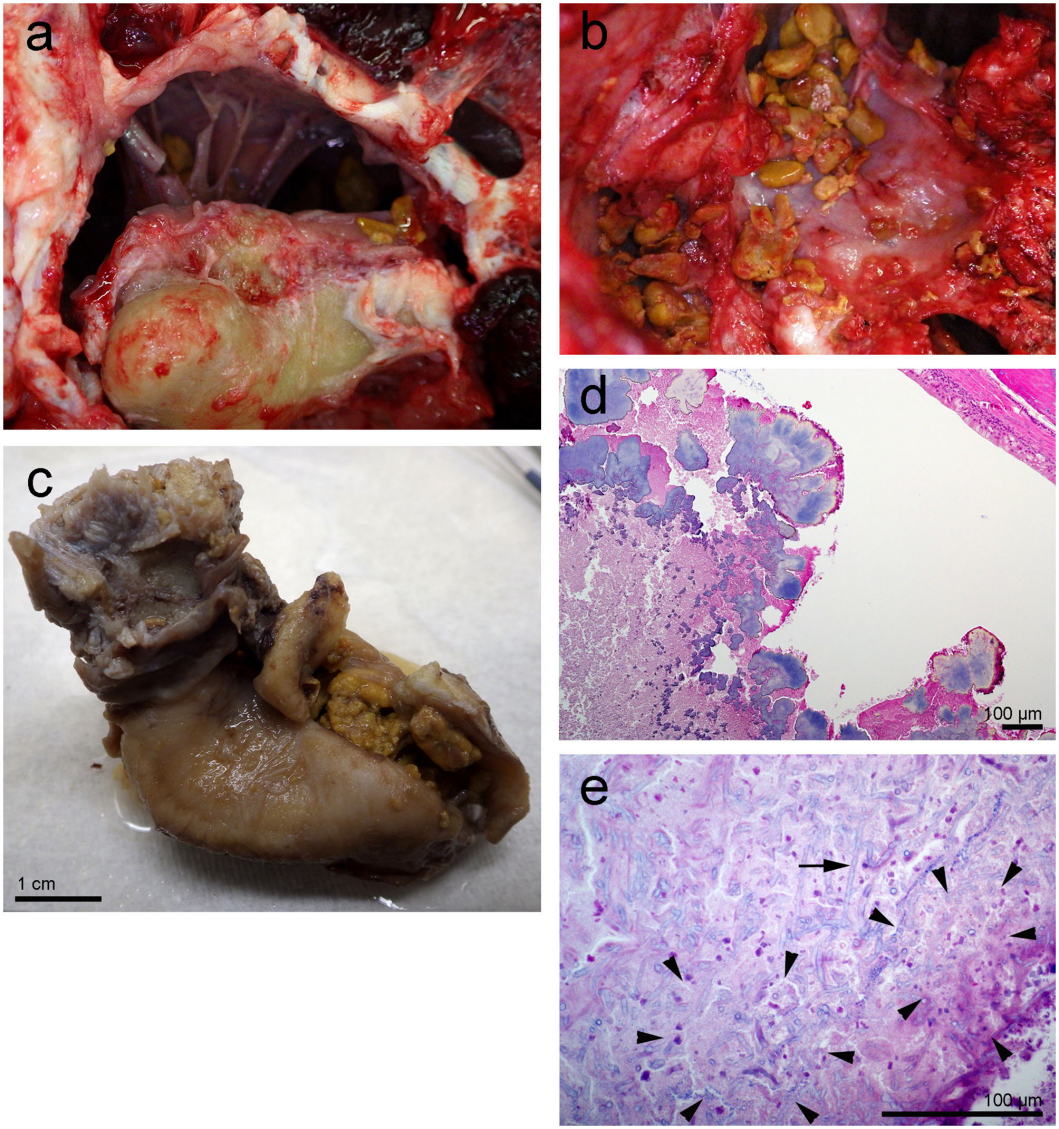
Left tympanic bone from the beluga Qila. The gross images, **(a,b)** were taken during the necropsy and dissection of the ears. Note the smooth nodular tan yellow precipitates in the peribullar **(a,b)** and tympanic cavities **(c)**. Image **(b)** was obtained after the removal of the tympano-periotic complex. **(d,e)** Histopathology of hematoxylin-eosin stained left tympanic bone and cavity with numerous lobules of bacteria **(d)** and florid fungal hyphae interspersed [arrow in **(e)**] within inflammatory infiltrate and necrotic debris [regions delimited by arrowheads in **(e)**].

On gross exam, the left tympanic cavity and the peribullar cavity contained numerous 0.1–0.3 cm diameter, tan yellow friable mineralized deposits (Figures 2a–c). Histopathology revealed large aggregates of fungal hyphae that were regularly septate, dichotomously branching, parallel and occasionally slightly dilated. These hyphae were morphologically consistent with *Aspergillus* sp. (arrow in Figure 2e). There was mild reactive change in the adjoining mucosa and multifocal dystrophic mineral deposition (Figures 2d,e). A morphologic diagnosis of pyogranulomatous cochleotitis (regions delimited by arrowheads in Figure 2e) with intralesional hyphae morphologically consistent with *Aspergillus* sp. (arrows in Figure 2e) was made. The impact of the fungal aggregate on hearing could not be determined based solely on histopathology.

**Figure 3:**
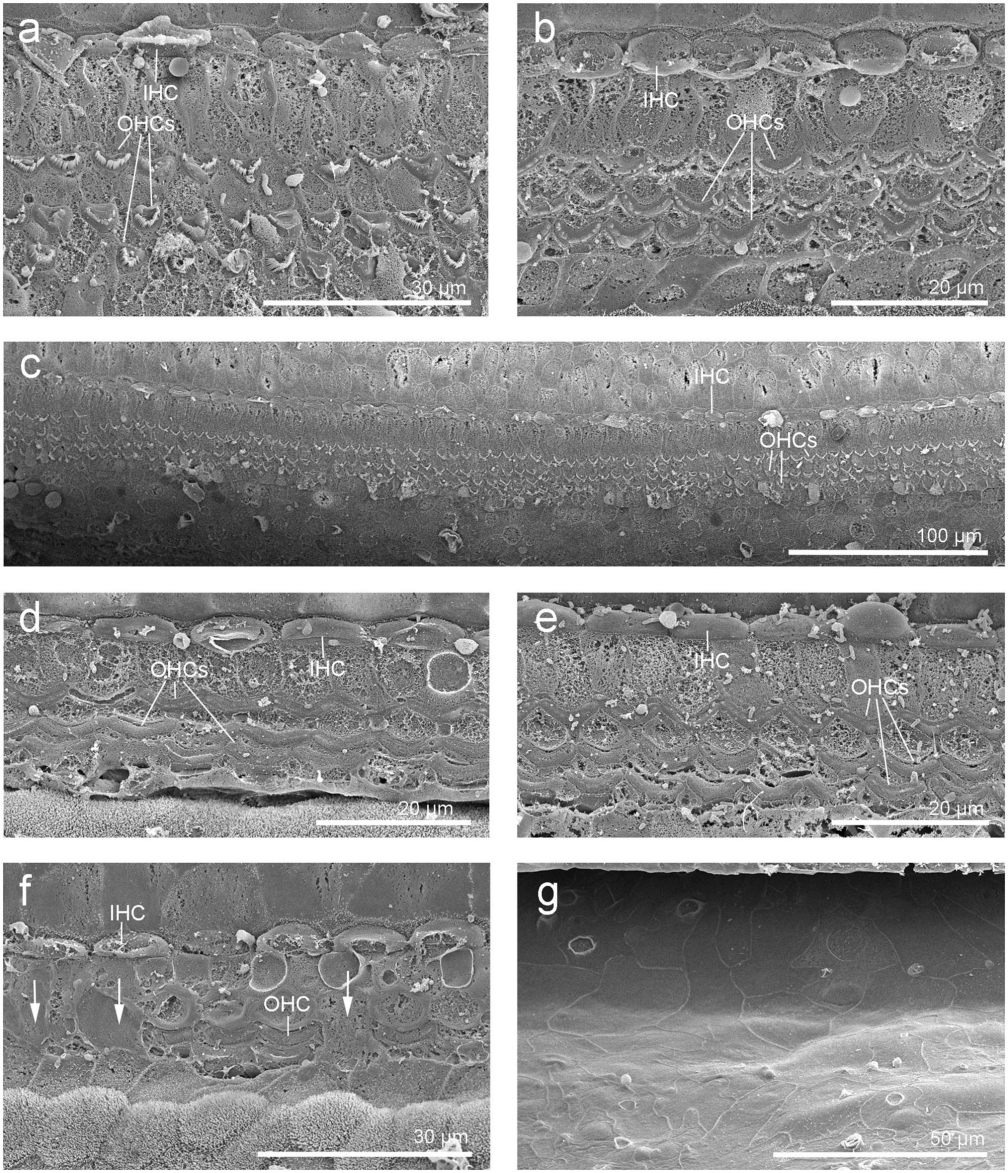
Scanning electron micrographs of the left cochlea from the beluga Qila. Organ of Corti with three rows of outer hair cells (OHCs) and one row of inner hair cells (IHCs) in the upper **(a)** and lower **(c)** apical turn, upper **(b)** and lower **(d–g)** basal turn. **(f)** Area of transition with some scars (arrows). **(g)** Flat epithelium, present along the first 4.9 mm of the base, where the highest frequencies are encoded.

SEM of the inner ear revealed that the cells of the organ of Corti were well preserved and the typical mammalian disposition of sensory cells, aligned as three rows of outer hair cells and single row of inner hair cells, was present throughout the cochlear spiral (Figure 3). However, in the first 4.9 mm from the base, which is the region where the highest frequencies of the hearing range are encoded, there was a well circumscribed “flat epithelium” consistent with a chronic and severe process and attributed to sensory cell death by apoptosis (Figure 3g). In this case, the specialized supporting cells had either dedifferentiated or were replaced by simple epithelial cells. The area of transition between the flat epithelium and the “normal” sensory epithelium was small, of ~400 μm, with a few missing hair cells (arrows in Figure 3f).

#### Right Inner Ear From Qila

**Figure 4:**
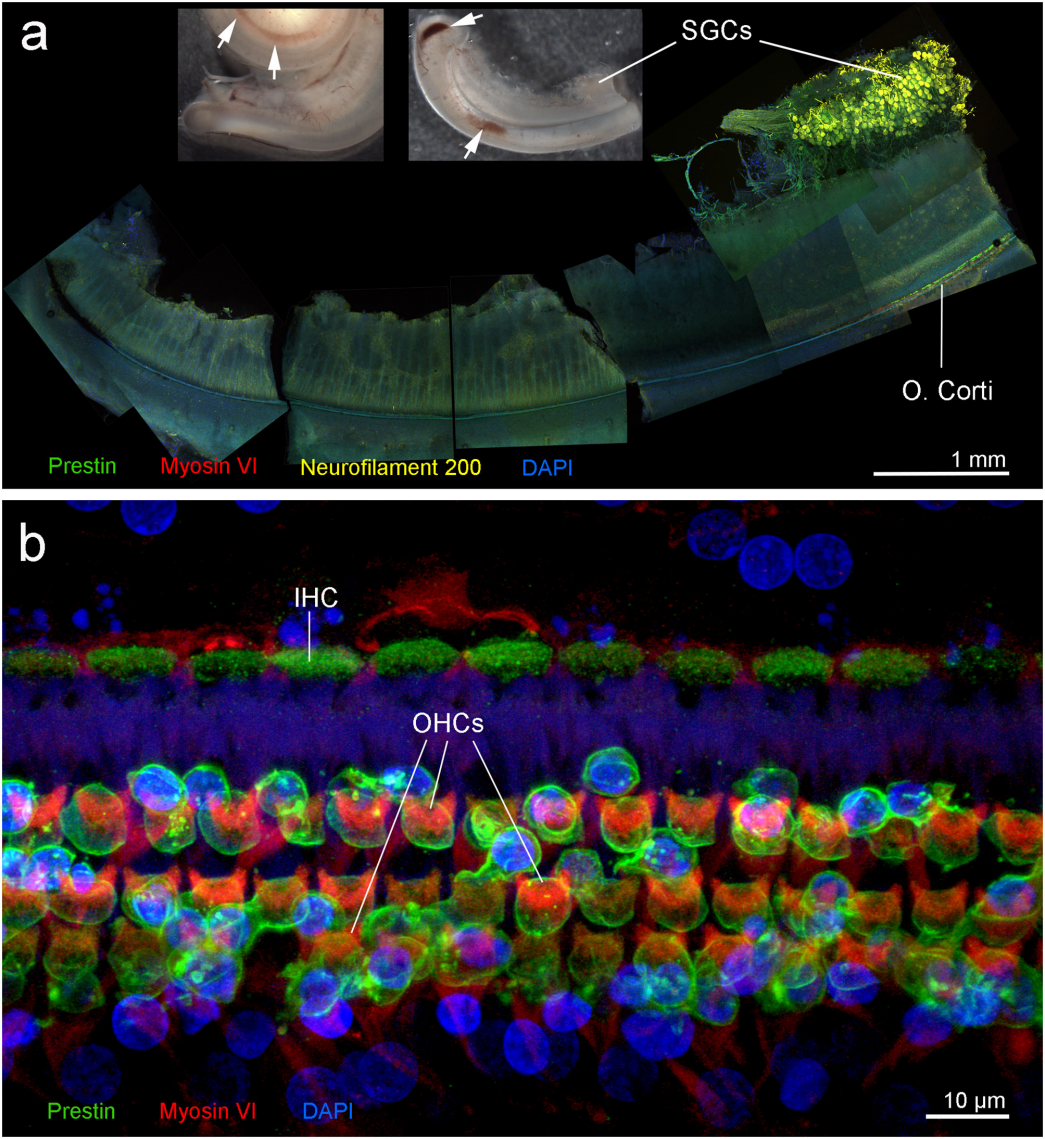
Right cochlea from the beluga Qila. **(a)** Sub-gross focal hemorrhage (arrows) and immunofluorescence image of the hook or most basal part of the cochlea, where the highest frequencies are encoded. **(b)** Organ of Corti (O. Corti) of the lower apical turn, formed by three rows of outer hair cells (OHCs) and one row of inner hair cells (IHCs). Flat preparations of the cochlea were incubated with antibodies against prestin (green), myosin VI (red), neurofilament 200 kD (yellow) and DAPI (blue). SGCs, spiral ganglion cells.

**Figure 5:**
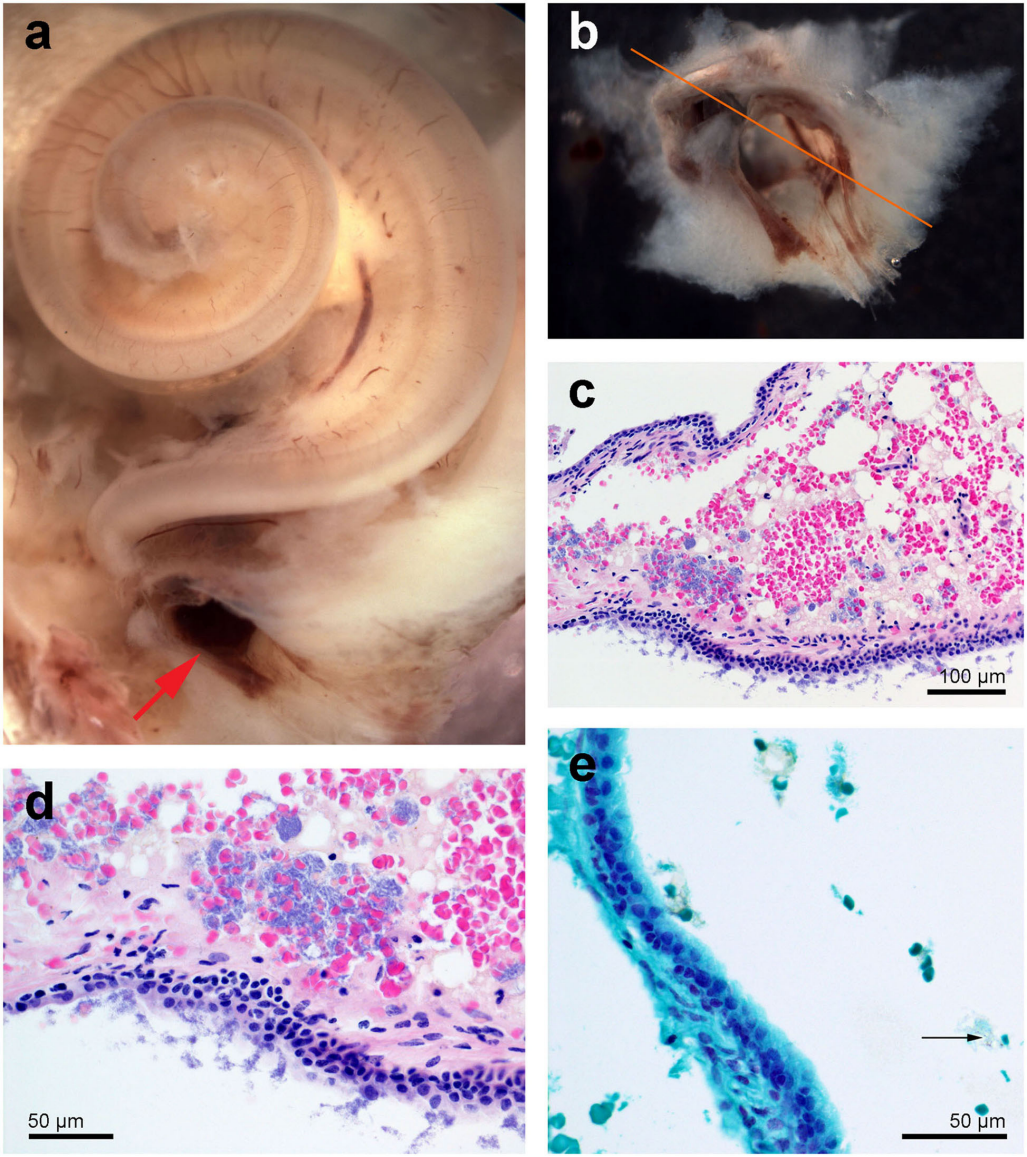
Right cochlea from the beluga Qila. **(a)** General picture. Note there is multifocal congestion with hemorrhage at the round window (red arrow). **(b)** Detail of the round window niche after being sampled for histopathology. **(c,d)** Histopathology of focally extensive acute hemorrhage admixed with vacuolated proteinaceous deposits and dense clusters of extracellular **(c,d)** and rare intracellular [arrow in **(e)**] Gram positive cocci. Sections **(c,d)** were stained with hematoxylin-eosin and **(e)** with Twort's gram stain.

During the dissection, moderate, multifocal acute hemorrhage was found at the end of the hook, lower apical turn, upper basal turn (arrows in Figure 4a) and in the round window (red arrow in Figure 5a). A source of the hemorrhage was not readily apparent on gross examination. The decalcified bone tissue around the round window as well as the round window niche and part of the tympanic scala were dissected and prepared for histopathology (Figure 5).

Histopathology of the round window niche and surrounding tissue confirmed acute hemorrhage with numerous intralesional Gram positive cocci (Figures 5c,d). Despite the lack of associated inflammatory infiltrate, the hemorrhage suggested peracute bacteremia and embolization, rather than *post mortem* bacterial overgrowth.

Immunofluorescence on the right ear showed no hair cells or type I afferent innervation in the first 6.6 mm of the base of the cochlea; whereas, the rest of the cochlea presented type I innervation and a typical organ of Corti formed by three rows of outer hair cells and one row of inner hair cells (Figure 4b). Due to unintentional damage during dissection, the upper apical turn of the organ of Corti could not be assessed by immunofluorescence. However, the innervation appeared within acceptable limits, suggesting that there was no evidence of hearing loss in this particular region. The three controls (specificity control, control for non-specific binding of the secondary antibodies, and autofluorescence control) were negative.

#### Left Inner Ear From Aurora

The sub-gross dissection disclosed mild hemorrhage in the lower basal turn. In addition, there were white caseous to friable irregular nodular deposits in the round window niche. The round window niche was sampled for histopathology while the rest of the cochlea was dissected for SEM evaluation.

**Figure 6:**
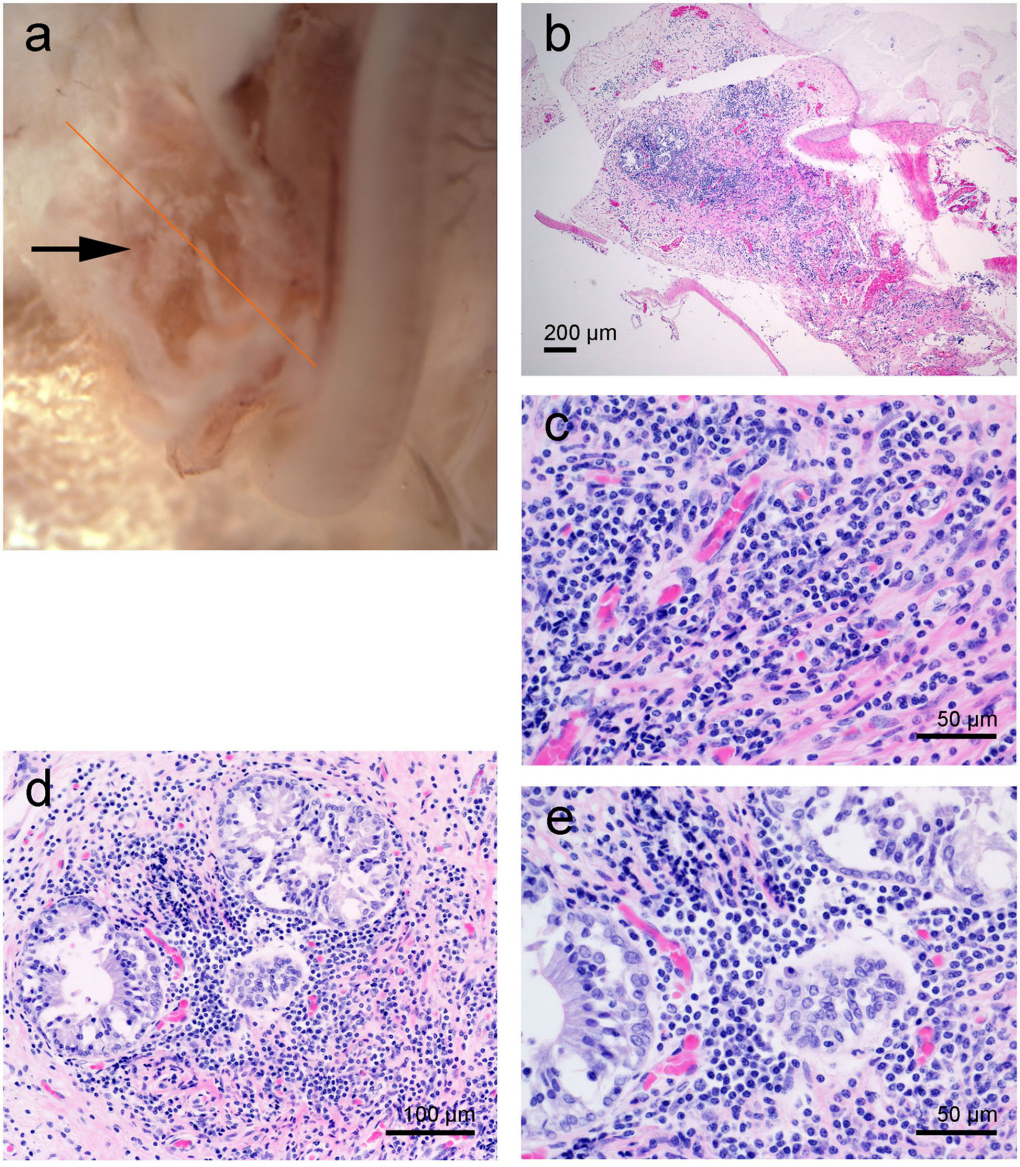
Left cochlea from the beluga Aurora. **(a)** Sub-gross image of the base of the cochlea. The arrow highlights the white deposits at the round window niche. **(b–d)** Micrographs of Hematoxylin and Eosin stained sections with moderate focal accumulation of lymphocytes, plasma cells and fewer macrophages with scattered neutrophils. In images **(d,e)** there are a small number of entrapped variably sized glands.

Histopathology revealed that within the lumen, at the base of the cochlea, there was a dense aggregate of inflammatory infiltrate comprised of lymphocytes and plasma cells and scattered neutrophils (Figure 6). Entrapped within the inflammatory infiltrate were a few variably sized glandular elements with vacuolated and occasionally artifactually detached epithelia (Figures 6c,d).

**Figure 7:**
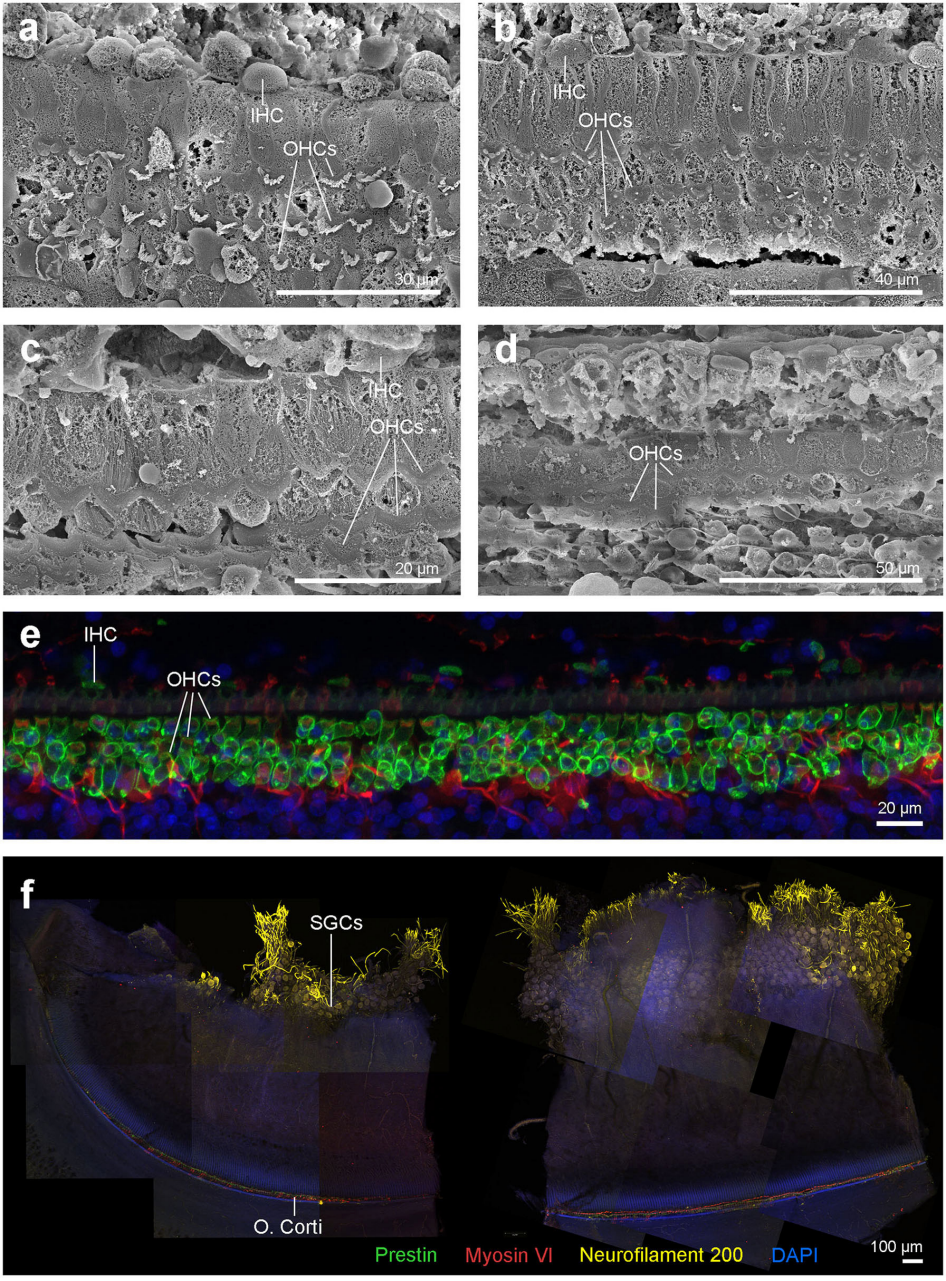
**(a–d)** Scanning electron micrographs of the left cochlea from the beluga Aurora. Organ of Corti features three rows of outer hair cells (OHCs) and one row of inner hair cells (IHCs) in the upper **(a)** and lower **(b)** apical turn, upper **(c)** and lower **(d)** basal turn. **(e,f)** Right cochlea from the beluga Aurora. Immunofluorescence images of the middle turn **(e)** and hook or most basal part of the right cochlea. **(f)** The beginning of the hook is located at the left the image, which is the region of the cochlea where the highest frequencies are encoded. Flat preparations of the cochlea were incubated with anti- prestin (green), anti-myosin VI (red), anti-neurofilament 200 kD (yellow) antibodies and DAPI (blue). SGCs, spiral ganglion cells.

SEM revealed that the cells of the organ of Corti in Aurora were less well preserved than in Qila and the discrepancy was attributed to a delay between death, ear collection and fixation with Aurora. Although three rows of outer hair cells and the single row of inner hair cells were distinguished throughout the cochlear spiral (Figures 7a–d), in a small region of the upper basal turn the organ of Corti was obscured by a processing artifact. In the most basal portion of the cochlea, the organ of Corti was folded. Although hair cells could not be evaluated ultrastructurally, the normal morphology of phalangeal processes of Deiters cells indicated that the outer hair cells were alive *ante-mortem*. In case of outer hair cell death, the phalangeal processes would have swollen to seal the reticular lamina, and this feature was not apparent in the examined cochlea. Based on SEM evaluation, there was no evidence of ultrastructural pathology or hearing loss in the left inner ear of this individual.

#### Right Inner Ear From Aurora

The right cochlea was sectioned flat and dissected using the whole-mount technique and prepared for immunofluorescence. A small region of the lower basal turn had mild hemorrhage in the vestibular scala, which was not apparent in the cochlear scala.

There was positive labeling for hair cells and associated innervation throughout the cochlear spiral (Figures 7e,f). Artifact associated with *post-mortem* decomposition was more evident in the upper basal and lower apical turns. The three controls were negative. Based on observation from immunofluorescence, there was no evidence of hearing loss at the level of the hair cells or innervation of the right inner ear.

## Discussion

Ultrastructural and immunofluorescence studies of both ears in Qila revealed lesions in the base of the cochlea consistent with bilateral high-frequency hearing loss in this individual. There was a slightly larger affected area in the right ear. AEPs measured 1.5 years prior to this beluga's death showed hearing loss, particularly at high frequencies of 56 kHz and above, and apparently more severe in the right ear. This was apparent in the low amplitudes and delayed latencies of the peaks of the ABR, which are associated with high-frequency hearing loss in cetaceans (33). The frequency-specific thresholds in the audiograms subsequently confirmed such a hearing loss. This beluga may have had additional pathology within its hearing pathway that could explain the generalized hearing impairment. Based on the AEPs and morphology studies, Qila would have had mixed hearing loss, which is a combination of conductive and sensorineural hearing loss.

Masking of hearing thresholds by ambient noise did not account for the elevation in Qila's hearing thresholds [see (34)]. Based on the ambient noise levels measured in the pool, and critical ratios published by Johnson et al. (30), hearing thresholds for Aurora between 20 and 56 kHz were 5–10 dB above the expected minimum thresholds in the presence of energetic masking. Aurora's thresholds were further elevated above these predictions at higher frequencies (22, 41, and 56 dB differences at 80, 113, and 130 kHz, respectively). Qila's even higher thresholds therefore resulted from a true deficit in auditory function rather than interference from ambient noise.

In domestic animals and humans, high-frequency hearing loss can be due to age related degeneration [i.e., presbycusis, (35)]; however, Qila was 21 years old while wild belugas have an estimated mean lifespan of 30–60 years (36–38). Another cause of similar lesions to the ones found in our study in the most basal region of the cochlea could be exposure to ototoxic drugs, as observed in a beluga by Finneran et al. (32). However, since there was no record of clinical history of ototoxic drug administration, it is unlikely that this may have contributed to the hearing loss in this case. Noise-induced hearing loss is frequency specific, although a high-frequency noise source able to damage this part of the cochlea would need to be present at a sufficiently high level and/or long duration in the belugas' environment (27, 39). Underwater noise measurements conducted at the Vancouver Aquarium in 2014 revealed no noise capable of inducing this hearing loss, and it is unlikely that there were other sources that emit at these frequencies in the vicinity of the display that the animal would be exposed to. In addition, Aurora would have likely been exposed to the same sound source since both whales lived in the same habitat and Qila was born at the Aquarium.

Other potential causes could include congenital, infectious, toxic or immunological disorders. However, the small zone of transition between the affected vs. non-affected area in the basal turn, as well as the fungal infection of the middle ear, suggests that ischemia and apoptosis may have resulted in the high-frequency hearing impairment. Studies in humans showed that vertebrobasilar artery ischemia can cause sensorineural hearing loss (40). In animal models, it was demonstrated that labyrinthitis induced a reduction of cochlear blood flow or ischemia, associated with hair cell loss (41). Temporal bone histopathological studies in humans showed that suppurative labyrinthitis resulted in stria vascularis atrophy, hair cell and auditory neuron loss, mainly in the basal turn of the cochlea explaining high-frequency hearing loss (42, 43). Altogether, these observations suggest that the pathological findings in Qila could be a consequence of a labyrinthitis related or secondary to chronic middle ear infection.

Conversely, the results of the inner ear analysis and AEP measurements in Aurora were different. Although there were some artifacts attributed to *post mortem* decomposition, both inner ears from the beluga Aurora were remarkably well preserved considering the delay between death, sample collection and fixation (16 h). At present, there is little information regarding the pathology and neurophysiology of the cochlea in belugas, and the repercussions of the chronic inflammatory process found in the round window niche on hearing are unknown. Based on analysis of the ultrastructure of the hair cells (SEM and immunohistochemistry) and associated afferent innervation (immunohistochemistry), there was no evidence of hearing loss in either ear. Our anatomical results are thus consistent with the AEPs measured for this individual.

It is unusual that two cetaceans in captivity die in a short time span (9 days apart). Although tragic, these mortalities afforded a unique opportunity to compare three methodologies and results obtained from two related individuals that shared the same environment for over 20 years with different hearing capabilities. Although the results of the post-mortem examination were different in both cases, both deaths were acute, and there is no clinical history, histopathologic or ultrastructural evidence that suggests that the cause of death was related with the hearing disorder found in Qila. The lack of detectable of type I afferent neurons and the presence of flat epithelium in the base of the cochlea can be an indication of an old lesion, and consistent with the results obtained with AEPs. In addition, we would have also found damage in the inner ear of Aurora if the lesions detected in Qila would have been related to the death of both individuals since Qila was a severe case. We do not expect that significant changes in the hearing capabilities of both individuals occurred between the measurement of their AEPs and their death and cochlear analysis 1.5 years later. There are detailed medical records for both animals in this time period. No ototoxic drugs were administrated, or clinical detection of infections that may have contributed to hearing loss in either of these individuals. Age-related hearing loss during this time period could be a factor to consider, but we would have likely detected it in Aurora as well, given that she was 19 years older than Qila.

Although we compared the three techniques on two individuals and statistical analysis could not be conducted due to the small dataset, the fact that comparable results were obtained with all three techniques used shows that physiological and morphological test results can be correlated. This is particularly important because it is rare to have information on the cochlear anatomy and AEPs of the same individual: access to live animals is necessary for the measurement of AEPs, and rapid preservation of the hearing organs following death is required for cochlear anatomical analyses. The comparison shown in our study will be also useful in those circumstances when only one of the two methods (AEPs or inner ear analysis) can be performed.

This study is the first to compare the findings of two cochlear analysis techniques and direct hearing sensitivity measurements from AEPs in a cetacean species. The ability to combine morphological and auditory data is crucial to validate cochlear findings in stranded cetaceans, as well as to validate predictions of cochlear frequency maps based on morphological features. Animals with hearing loss are of particular interest since they can further refine predictive models, and cases of high-frequency hearing loss will be crucial to localize the frequencies encoded in the first millimeters of the base of the cochlea. While AEPs on live strandings are a valuable tool to assess the hearing capabilities of species that cannot be kept in captivity (44), cochlear frequency maps will allow for predictions of the full hearing range of species for which it is not possible to measure AEPs *in vivo*, or where such data are not yet available. Further, studies on noise-induced hearing loss in terrestrial and marine mammals exposed to high noise levels show that the greatest losses of sensitivity regularly occurs at a frequency about half an octave, up to one octave, above the exposing tone (39, 45–47). Thus, in future cases of noise-induced hearing loss in individual and mass stranded marine mammals, frequency maps could ultimately provide key information on the frequency characteristics of the causal sources of the cochlear lesions.

## Data Availability Statement

The original contributions presented in the study are included in the article, further inquiries can be directed to the corresponding author/s.

## Ethics Statement

The animal study was reviewed and approved by the Vancouver Aquarium Institutional Animal Care and Welfare Committee. The ears from belugas were transported with the appropriate CITES permits.

## Author Contributions

MM performed the inner ear extraction, dissection, their preparation for confocal and electron microscopy, and image interpretation. SR conducted the necropsies and histopathological evaluation of the tissues. JM measured the auditory evoked potentials in both belugas. MH, LB-L, and CN were in charge of the care of the belugas and associated research programs. FV helped with the evaluation of the results. MM, SR, JM, MH, LB-L, CN, FV, and RS helped with writing and editing the manuscript. RS and SR supervised the work and its publication. All authors contributed to the article and approved the submitted version.

## Conflict of Interest

The authors declare that the research was conducted in the absence of any commercial or financial relationships that could be construed as a potential conflict of interest.
